# New Insights Into the Role of Ca_v_2 Protein Family in Calcium Flux Deregulation in *Fmr1*-KO Neurons

**DOI:** 10.3389/fnmol.2018.00342

**Published:** 2018-09-27

**Authors:** Sara Castagnola, Sébastien Delhaye, Alessandra Folci, Agnès Paquet, Frédéric Brau, Fabrice Duprat, Marielle Jarjat, Mauro Grossi, Méline Béal, Stéphane Martin, Massimo Mantegazza, Barbara Bardoni, Thomas Maurin

**Affiliations:** ^1^Université Côte d’Azur, CNRS UMR7275, IPMC, Valbonne, France; ^2^CNRS LIA “Neogenex”, Valbonne, France; ^3^Université Côte d’Azur, INSERM, CNRS UMR7275, IPMC, Valbonne, France

**Keywords:** Fragile X syndrome, Ca_v_2.1, calcium homeostasis, ratiometric calcium imaging, *Cacna1a*

## Abstract

Fragile X syndrome (FXS), the most common form of inherited intellectual disability (ID) and a leading cause of autism, results from the loss of expression of the *Fmr1* gene which encodes the RNA-binding protein Fragile X Mental Retardation Protein (FMRP). Among the thousands mRNA targets of FMRP, numerous encode regulators of ion homeostasis. It has also been described that FMRP directly interacts with Ca^2+^ channels modulating their activity. Collectively these findings suggest that FMRP plays critical roles in Ca^2+^ homeostasis during nervous system development. We carried out a functional analysis of Ca^2+^ regulation using a calcium imaging approach in *Fmr1*-KO cultured neurons and we show that these cells display impaired steady state Ca^2+^ concentration and an altered entry of Ca^2+^ after KCl-triggered depolarization. Consistent with these data, we show that the protein product of the *Cacna1a* gene, the pore-forming subunit of the Ca_v_2.1 channel, is less expressed at the plasma membrane of *Fmr1*-KO neurons compared to wild-type (WT). Thus, our findings point out the critical role that Ca_v_2.1 plays in the altered Ca^2+^ flux in *Fmr1*-KO neurons, impacting Ca^2+^ homeostasis of these cells. Remarkably, we highlight a new phenotype of cultured *Fmr1*-KO neurons that can be considered a novel cellular biomarker and is amenable to small molecule screening and identification of new drugs to treat FXS.

## Introduction

Fragile X syndrome (FXS) is the most common form of inherited intellectual disability (ID) and the leading identified monogenic cause of autism (Maurin et al., 2014; Castagnola et al., 2017). FXS is caused by the silencing of the *Fmr1* gene encoding the Fragile X Mental Retardation Protein (FMRP), an RNA-binding protein modulating the expression of thousands of mRNAs primarily at the translational level in particular, it has been shown to regulate translation at the synaptic level. Furthermore, FMRP has been reported to be involved in different steps of RNA metabolism, indeed it is a component of various ribonucleoproteic complexes (mRNPs), including the RNA granules, the mRNP involved in transport along neurites (Maurin et al., 2014, 2018a).

Several reports have shown that FMRP binds multiple RNAs encoding regulators of ion homeostasis and more particularly involved in the calcium ion pathway (Brown et al., 2001; Miyashiro et al., 2003; Darnell et al., 2011; Ascano et al., 2012; Maurin et al., 2018a). Furthermore, the search for FMRP-interacting proteins has resulted into the identification of dozens of partners, including ion channels (Bardoni et al., 2006; Ferron, 2016; and this study). Consistently with these findings, ion homeostasis defects in FXS neurons have been described (Chen et al., 2003; Meredith et al., 2007; Brown et al., 2010; Deng et al., 2013; Ferron et al., 2014; Hebert et al., 2014; Zhang et al., 2014; Contractor et al., 2015; Myrick et al., 2015; Wahlstrom-Helgren and Klyachko, 2015; Achuta et al., 2018). In particular, FMRP has been reported to directly interact with two members of the Voltage Gated Calcium Channels (VGCC) family, namely Ca_v_2.1 and Ca_v_2.2 (Ferron et al., 2014).

Cytosolic calcium concentration is set by the balance between calcium influx and efflux as well as by the exchange of calcium ion with internal stores. Calcium homeostasis is tightly controlled and involves multiple protein complexes such as ATPase pumps, transporters and ion channels in various cellular compartments (Clapham, 2007).

VGCCs respond to plasma membrane depolarization by allowing extracellular calcium ions to flow into cells according to their concentration gradient. Calcium can then act as a second messenger of cell depolarization activating various key intracellular signaling pathways, inducing contraction in muscle cells, protein phosphorylation, secretion and synaptic transmission. VGCCs are heteromers composed by the assembly of a pore-forming subunit (encoded by the corresponding α1 gene) and auxiliary ß and α2∂ proteins (Dolphin, 2016). VGCCs can be distinguished as L-, N-, R- and P/Q-type channels depending on the identity of the pore-forming subunit. L- and T-type VGCCs are found in a great variety of cells, while N-, P/Q- and R-type are mostly expressed in neurons (Catterall, 2011).

The *Cacna1a* gene encodes the P/Q-type VGCC Ca_v_2.1, which is critical for the depolarization-evoked release of neurotransmitters at the presynaptic terminals (Simms and Zamponi, 2014). Ca_v_2.1 is mostly expressed in the cerebellum, consequently mutations in the *Cacna1a* gene are associated with several neurological disorders such as episodic ataxia and spino-cerebellar ataxia (Zhuchenko et al., 1997). More recently, new mutations in this gene have been identified in four unrelated families with ID, attention deficit, hyperactivity and autism spectrum disorder (Damaj et al., 2015). This suggests that Ca_v_2.1 may play a previously under-appreciated role in brain regions other than the cerebellum and could have be implicated roles in cognition, memory and social interaction regulation. Indeed, regulation of Ca_v_2.1 channels by calcium sensor proteins is required for normal short-term synaptic plasticity, LTP, and spatial learning and memory in mice (Nanou et al., 2016).

We thus investigated calcium homeostasis using ratiometric calcium imaging in *Fmr1*-KO neurons. Our results show that neurons lacking FMRP are not only more sensitive to Ca_v_2.2 inhibition but also less sensitive to Ca_v_2.1 inhibition compared to wild-type (WT) neurons and this is a consequence of an impaired membrane expression of this channel in the absence of FMRP. We propose here a model in which FMRP is involved in the regulation of the relative membrane expression of P/Q- and N-type VGCCs.

## Materials and Methods

### Primary Neuronal Cultures

Cultures were prepared from the cortex of embryonic stage E14.5 WT and *Fmr1*-KO embryos as previously described (Abekhoukh et al., 2017; Maurin et al., 2018a). Neurons (250,000 cells) were plated on ornithine-coated glass coverslips (35 mm diameter) and cultivated in complete medium: Neurobasal (Invitrogen) supplemented with B-27 (Invitrogen) and glutamax (Invitrogen). Neurons were fed weekly by removing 10% of the culture medium and replacing it with fresh complete medium.

### Ratiometric Calcium Imaging

Primary cortical neurons Day-*In-Vitro* 19–23 (DIV 19–23; 13 independent cultures) grown on coverslips were incubated in neurobasal containing 20 μM Fura2-AM (Invitrogen) for 30 min at 37°C. After two washes with HEPES-buffered Tyrode’s calcium solution (in mM: 139 NaCl, 15 glucose, 1.25 Na_2_HPO_4_ dibasic heptahydrate, 1.8 MgSO_4_ heptahydrate, 1.6 CaCl_2_ dihydrate, 3 KCl, 10 HEPES), coverslips were placed in a metal chamber on an inverted microscope (AxioObserver, Carl Zeiss) equipped with a 300W Xenon lamp (Sutter Instruments) and a Fluar 40× NA 1.4 oil immersion objective. Cells were perfused at 22°C throughout the recording with Tyrode’s calcium solution. The pharmacological stimulations were performed by supplementing the calcium recording solution with either DiHydroxyPhenylGlycine (DHPG, 100 μM) or KCl (50 mM) or VGCC antagonist (ω-agatoxin-Iva (100 nM); ω-conotoxin GVIa (1 μM); Nitrendipine (1 μM)) or VGCC antagonist (same concentrations) + KCl. A calibration step was performed at the end of every recording by applying successively 0 Ca^2+^ (in mM: 129 NaCl, 15 glucose, 1.25 Na_2_HPO_4_ dibasic heptahydrate, 1.8 MgSO_4_ heptahydrate, 0.5 EGTA, 3 KCl, 10 HEPES), then 0 Ca^2+^ + ionomycin (5 μM) and finally 10 Ca^2+^ + ionomycin (5 μM; in mM: 129 NaCl, 15 glucose, 1.25 Na_2_HPO_4_ dibasic heptahydrate, 1.8 MgSO_4_ heptahydrate, 10 CaCl_2_ dihydrate, 3 KCl, 10 HEPES) solutions. This calibration step allows to quantify the lowest and the highest probe fluorescence F_340/380_ ratio for every Region of Interest (ROI); the maximal value was used subsequently to normalize the fluorescence F_340/380_ measurements. Every recording experiment followed the same protocol:

Tyrode’s—40 s; Tyrode’s + DHPG—40 s; Tyrode’s—60 s; Tyrode’s + KCl—20 s; Tyrode’s—60 s; Tyrode’s + KCl—20 s; Tyrode’s—60 s; Tyrode’s + VGCC antagonist—60 s; Tyrode’s + KCl + VGCC antagonist—20 s; Tyrode’s + VGCC antagonist—60 s; Tyrode’s + KCl + VGCC antagonist—20 s; Tyrode’s + VGCC antagonist—60 s; Tyrode’s + KCl + VGCC antagonist—20 s; Tyrode’s + VGCC antagonist—60 s; Tyrode’s (0 Calcium)—80 s; calibration (see above).

Fura2 was sequentially excited at 340 nm and 380 nm, and the emission monitored at 510 nm. Images were acquired with a cascade 512 EMCCD camera every 2 s using the Metafluor software (Roper Scientific). For each recorded cell, the intracellular calcium concentration [Ca^2+^]_i_ was estimated by measuring the F_340/380_ nm ratio of fluorescence normalized to the maximal probe fluorescence measured when cells were perfused with the 10 Calcium + ionomycin solution. ω-agatoxin-IVa and ω-conotoxin GVIa were purchased from Smartox, Nitrendipine from Sigma-Aldrich. Resting calcium levels (“baseline”) were measured as the average fluorescence from the first 40 s of each recording. For KCl stimulation, for each cell analyzed we report the results of the mean of two maximal F_340/380_ in two consecutive stimulations. The Drug Response (DR) represents the mean of the two max F_340/380_ in two consecutive stimulations over the mean of the three max F_340/380_ in three consecutive stimulations in the presence of antagonist. The results of the pharmacological stimulations (DHPG, KCl) are reported as fold change over baseline levels. Only cells for which the DHPG stimulation elicited a fold change greater than 1.1 times the baseline levels in F_340/380_ ratio were considered responsive cells.

### Immunoprecipitation

Cerebella from WT and *Fmr1*-KO mice were grinded in liquid nitrogen into fine powder and resuspended in 5 v/w with PBS containing 1% Igepal. Samples were cleared with 15 μl of naked Dynabeads A (Thermofisher) for 30 min at 4°C on a rotating wheel. During this time, 30 μl of Dynabeads A were incubated with anti-FMRP primary antibody for 1 h at room temperature on a rotating wheel, with 100 μg of tRNA, ssDNA and BSA. The “pre-clear” beads were then removed and samples were centrifuged for 10 min at 14,000 rpm at 4°C. Supernatants were incubated with antibody-coated beads overnight at 4°C on a rotating wheel. Beads were washed three times with PBS containing 0.1% Igepal and incubated for 15 min at 55°C with 100 mM dithiothreitol and 2× Laemmli sample buffer. Eluted proteins were then resolved on 4%–12% gradient SDS–PAGE using MOPS buffer (Invitrogen).

### Biotinylation

Primary neurons plated at the density of 200,000 cells per well were used for biotinylation experiments at DIV 15. Neurons were washed twice with PBS and incubated with EZLink Sulfo-NHS-LC-Biotine (0.3 mg/ml in PBS, Thermo Scientific) for 10 min at 4°C. After a quick wash with PBS, unbound biotin molecules were quenched with 50 mM NH_4_Cl for 5 min. After two washes with ice-cold PBS, proteins were extracted using lysis buffer containing 10 mM Tris-HCl pH 7.5, 10 mM EDTA, 150 mM NaCl, 1% Triton X-100, 0.1% SDS and 1% mammalian protease inhibitor cocktail (Sigma-Aldrich). Two-hundred microgram of proteins from each condition were incubated overnight at 4°C with streptavidin-conjugated beads (Sigma-Aldrich). Beads were then washed three times with lysis buffer and resuspended in Laemmli buffer. Proteins were separated in 7% acrylamide-bis-acrylamide gel. Primary antibodies anti ß-Actin (Sigma, #A5441; 1/1,000), anti-Ca_v_2.1 (Alomone Labs, #ACC-001; 1/1,000) and anti ß3-tubulin (Synaptic Systems, #302302; 1/1,000) were used.

RNA extraction and RT-qPCR were performed as previously described (Maurin et al., 2018a). The sequences of the primers used in this study are provided in Table 1.

**Table 1:** Sequences of the primers used in this study.

	Forward	Reverse
***Cacna1a***	GAGTATGACCCTGCTGCCTG	TGCAAGCAACCCTATGAGGA
***Cacna1b***	TGCGTTCTCGAGCTTCATGG	CGCTTGATGGTCTTGAGGGG
***Cacna1c***	GAACCATATCCTAGGCAATGCAG	AAGAGCCCTTGTGCAGGAAA
***Cacna1e***	TGAGTTTGTCCGTGTCTGGG	GAGGGACATCTCTTGCCGAG
***c-Kit***	GGAGTGTAAGGCCTCCAACG	TGGGCCTGGATTTGCTCTTT
***Klf4***	CAGGATTCCATCCCCATCCG	TGGCATGAGCTCTTGATAATGGA
***Gfap***	CAGATCCGAGGGGGCAAA	TGAGCCTGTATTGGGACAACT
***Dlg4***	GGCGGAGAGGAACTTGTCC	AGAATTGGCCTTGAGGGAGGA
***Tbp***	AGGCCAGACCCCACAACTC	GGGTGGTGCCTGGCAA

*Sequences are presented from 5’ to 3’ end*.

### Polyribosome Fractionation

Samples from polyribosome fractionation were described previously (Maurin et al., 2018a). Polyribosome fractionation was performed as described previously (Bechara et al., 2009) on 20%–50% (w/w) continuous sucrose gradients. Fractions were separated on a BR-188 Density Gradient Fractionation System (Brandel). Fold changes in *Cacna1a* mRNA levels between WT and *Fmr1*-KO were assessed by RT-qPCR and were calculated for individual fractions 6–14 according to the formula 2^−ddCp^ where ddCp is (Cp *Pde2a* KO fraction_x_–Cp *Gapdh* KO fraction_x_) – (Cp *Pde2a* WT fraction_x_–Cp *Gapdh* WT fraction_x_). Results from fractions 6 to 8 (light), 9 to 11 (medium) and 12 to 14 (heavy) were pooled and analyzed together.

### Protein Extraction and Western Blot Analysis

Cells and tissues extracts were processed as described previously (Maurin et al., 2018a). Primary antibodies anti ß-Actin (Sigma, clone AC-74; 1/1,000) and anti-Ca_v_2.1 (Alomone Labs, #ACC-001; 1/1,000) were incubated overnight at 4°C in PBS 0.05%.

### Immunocytochemistry on Primary Neurons

Primary neurons grown on glass coverslips were washed three times with PBS at room-temperature and then fixed using 4% Paraformaldehyde (PFA) in PBS for 10 min at room temperature. After rinsing briefly with PBS, free aldehydes were blocked with 50 mM NH_4_Cl in PBS for 5 min. Then, a saturation step was performed with PBS containing 10% Fetal Bovine Serum and 0.1% Triton X-100 for at least 20 min. Neurons were incubated with antibodies diluted in PBS containing 10% Fetal Bovine Serum and 0.1% Triton X-100 in a humidified chamber overnight at 4°C. After three PBS washes, neurons were incubated with secondary antibodies for 1 h at RT. After three PBS washes cells were incubated for 3 min in a PBS solution containing DAPI (10 μg/ml). The glass coverslips were finally washed once with ddH_2_O and mounted (Dako Fluorescent Mounting Medium) on glass slides and stored in the dark at 4°C. The polyclonal anti-Ca_v_2.1 (Alomone Labs, #ACC-001) antibody was used at a dilution of 1/50. The 1C3 antibody against FMRP was used at a dilution of 1/200 (Castets et al., 2005). Colocalization quantifications of FMRP and Ca_v_2.1 in one confocal plan (average of three scans) were carried out using the JACoP plugin for ImageJ (Bolte and Cordelieres, 2006). Cells were examined on a TCS SP5 confocal microscope (Leica).

### Cell Shape Analysis

We designed an ImageJ (Schneider et al., 2012) dedicated macro to analyze simultaneously the cell shape and the Fura2 fluorescence ratio variations (in time) obtained by sequential excitation at 340 and 380 nm. First, kinetics images of 340 and 380 nm excitation were stacked together and any lateral drift was corrected using the StackReg plugin (Thévenaz et al., 1998). A mask and a list of ROIs for each cell was obtained on the last 340 nm image after a filtering (recursive TopHat followed by an unsharp mask) and a Huang intensity thresholding. Then the 340 and 380 nm images were separated in two stacks and their F_340/380_ ratio calculated after a background measurement and subtraction in each image of the stack. The ROIs were then used on the 340/380 stack to get individual cell measurements of shape parameters (Aspect Ratio, Roundness, Area, Solidity) and F_340/380_ fluorescence ratios during time.

### Multivariate Analysis of the Cell Morphology Parameters

Baseline and KCl data were extracted and normalized to the maximal calcium value obtained for each cell with the 10 mM Calcium + ionomycin solution and combined to cell morphology parameters extracted from the images. Both cell morphology, normalized baseline and KCl data were then used for unsupervised analysis. Data were first log10 transformed, then mean-centered and scaled. Then, dimension reduction was performed using Barnes-Hut implementation of t-Distributed Stochastic Neighbor Embedding (tSNE), with perplexity parameter set to 40. K-means clustering was performed on the two-dimension tSNE projection and the optimal number of clusters was determined using the Gap statistic. Significance of the differences between continuous variable distributions was assessed using either Mann-Whitney or Kruskal-Wallis rank sum tests as appropriate. All analyses and graphical representations were performed using the R statistical package or Prism Software 6-2 version (GraphPad Software, Inc., San Diego, CA, USA).

### Statistics

The Kolmogorov-Smirnov test was used to assess the normality of the distribution of the datasets. To compare non-normally distributed data, two non-parametric tests were used: the Mann-Whitney test was applied to data of two unpaired samples, while the Kruskal-Wallis test was used to examine the significance of four unpaired groups. Data are expressed as mean ± SEM, and the *P* values (or adjusted *P* values) < 0.05 were considered statistically significant. RT-qPCR analysis of mRNA expression were analyzed using ANOVA TWO WAY with Sidak’s multiple comparisons *post hoc* test. The statistical analysis was performed using Prism Software 6-2 version (GraphPad Software, Inc.).

### Animal Experiments

The experiments were performed following the ARRIVE (Animals in Research: reporting *in vivo* Experiments) guidelines (Kilkenny et al., 2010). Animal care was conducted in accordance with the European Community Directive 2010/63/EU. The experiments were approved by the local ethics committee (Comité d’Ethique en Expérimentation Animale CIEPAL-AZUR N. 00788.01; APAFIS#4985-2016032314169426 v4APAFIS#8100-2016112217148206 v3).

## Results

### Calcium Homeostasis Is Impaired in *Fmr1*-KO Cells

We investigated calcium homeostasis using Fura2 ratiometric imaging in primary neuron cultures derived from the cortex of E14.5 WT and *Fmr1*-KO embryos. According to our immunocytochemistry results, these cultures are enriched in neurons and have limited mature astrocyte content (less than 10% of cells) that are mostly present in cell aggregates (Supplementary Figures S1A,B). Therefore, these regions were avoided in subsequent calcium recordings. RT-qPCR analysis of the expression of GFAP and PSD95 markers showed that the absence of FMRP does not affect the relative amounts of astrocytes and neurons in *Fmr1*-KO cultures compared to WT (Supplementary Figure S1C). We systematically applied a series of consecutive drug treatments followed by a calibration step that allowed us to quantify the minimum and maximum fluorescence of Fura2 in each analyzed cell. We used the normalized fluorescence ratio ([F_340/380_]/max[F_340/380_]) as an indirect quantification of the actual intracellular calcium concentration. By this imaging approach we investigated the functionality of several key parameters of calcium homeostasis in neurons in the presence or in the absence of FMRP.

### Cellular Analysis

**Figure 1:**
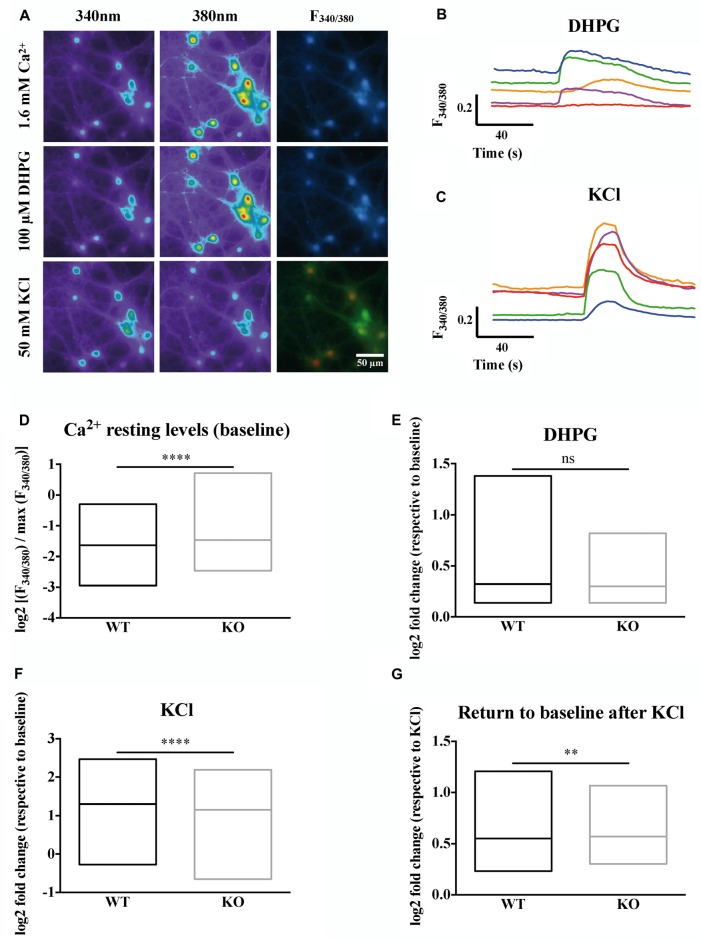
Calcium homeostasis is deregulated in Fragile X mental retardation 1-knockout (*Fmr1*)-KO neurons. **(A)** Profiles of the ratiometric calcium imaging response. Left panels show the emission of Fura2 at 340 nm. Middle panels show the emission of Fura2 at 380 nm. Right panels show the 340 nm/380 nm ratio of fluorescence (F_340/380_). Upper panels show the emission of Fura2 upon 1.6 mM Ca^2+^ perfusion. Middle panels show the emission of Fura2 upon 100 μM DiHydroxyPhenylGlycine (DHPG) perfusion. Lower panels show the emission of Fura upon 50 mM KCl perfusion. The scale bar of each panel is 50 μm. **(B)** Sample traces of Fura2 recording upon metabotropic glutamate receptor stimulation with DHPG (100 μM) or **(C)** depolarization with KCl (50 mM) in wild-type (WT) cells. For each cell recorded, the Fura2 fluorescence at each time was normalized to the maximum Fura2 fluorescence ratio observed in the presence of a solution containing 10 mM CaCl_2_ and ionomycin (5 μM). The mean stabilized F_340/380_ ratio of Fura2 fluorescence during the first 40 s of recording in the absence of any stimulation is represented in **(D)**. The log2 fold change in normalized F_340/380_ after 100 μM DHPG stimulation over baseline normalized ratio is presented in **(E)**. The log2 fold change in normalized F_340/380_ after 50 mM KCl stimulation over baseline normalized ratio is presented in **(F)**. The return to baseline following a KCl stimulation is shown for WT and *Fmr1*-KO neurons **(G)**. Mann-Whitney test: ****P < 0.0001; **P < 0.005; ns: *P* = 0.9963, not significant. WT*n* = 697; KO*n* = 744. These results are summarized in Table 2.

Our imaging data clearly show the heterogeneity of the neuronal types present in primary neuron cultures (Figures 1A–C). Cells differ in size, shape, resting intracellular calcium levels and maximum calcium entry upon KCl stimulation. We wondered whether the absence of FMRP could have different impacts on calcium homeostasis in different cell types. The Fura2 fluorescence ratio and the shape analysis of the ROIs were simultaneously quantified by an ImageJ lab-made macro giving the shape descriptors for each ROI (area, roundness, solidity, circularity). Roundness reflects how circular a ROI is, while solidity and circularity indicate how soft (high scores) or rough (low scores) are the contours of the region. We then performed an unsupervised multivariate analysis (Supplementary Figures S2A,B) to group cells according to their size, shape and calcium homeostasis parameters (baseline levels, maximum calcium levels upon KCl stimulation) identifying four distinct and homogeneous groups of cells (Supplementary Figures S2C–H). Representative images of ROIs detected in each cluster are shown in Supplementary Figure S3. Cells in group 1 and 3 differ in size and in the complexity of their contour, have a higher resting calcium concentration and high calcium entry upon KCl stimulation. Group 2 cells are small with rough contours and display a limited calcium entry following KCl stimulation, characteristics that suggest an astrocytic lineage. Group 4 ROI are small elongated objects that mostly correspond to neurites (Supplementary Figure S3). We considered the repartition of WT and *Fmr1*-KO cells in these clusters and our results indicate a homogeneous distribution of cells from the two genotypes in all clusters (Supplementary Figures S2I–L). The number of DHPG-responding cells was also similar in both genotypes (Supplementary Figure S4). We focused our analysis on cells belonging to group 1 and 3 which according to this analysis, have neuron characteristics. These cells were subsequently analyzed together. The steady state intracellular Ca^2+^ concentration, measured prior to any pharmacological treatment during the first 40 s of the recording, is elevated in the absence of FMRP (Mann-Whitney test, *P* < 0.0001; Figure 1D).

The metabotropic Glutamate receptor pathway has been described to be deregulated in FXS (Huber et al., 2002; Bear et al., 2004). The activation of this pathway with pharmacological agonists like DHPG triggers calcium release from internal stores through IP3 receptors as a consequence of the activation of the Phospholipase C and IP3 second messenger pathway. The calcium ion release from intracellular stores in response to DHPG is variable and not significantly different in the absence of FMRP compared to WT cells at the population level (Mann-Whitney test, *P* = 0.9963, not significant; Figure 1E).

We next induced cell depolarization by applying a 50 mM KCl solution onto the cultures, as in these conditions VGCCs are the main determinants of calcium entry in neurons (Mao et al., 2001). VGCCs respond to cell depolarization, upon which they open and allow calcium ion entry through their pore-forming subunit. We thus analyzed for each cell the fold change in F_340/380_ induced by KCl over baseline levels. Our results show that calcium entry through voltage-dependent plasma membrane channels upon KCl-induced neuron depolarization is slightly decreased in *Fmr1*-KO neurons (Mann-Whitney test, *P* < 0.0001; Figure 1F). Last, we observed that after the KCl stimulations *Fmr1*-KO neurons had significantly higher mean F_340/380_ ratio over the 40 s that followed the KCl stimulation compared to WT, suggesting a deregulated return to baseline levels in the absence of FMRP (Mann-Whitney test, *P* < 0.005; Figure 1G).

**Figure 2:**
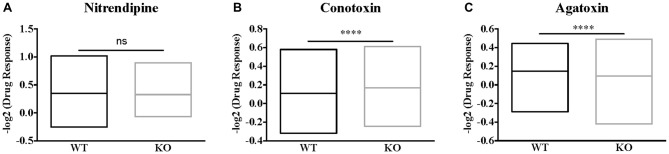
Voltage gated calcium channels (VGCC)-specific pharmacological approach reveals a decreased P/Q channel sensitivity to ω-Agatoxin IVa in *Fmr1*-KO cells. **(A)** Quantification of the drug response (DR; normalized max F_340/380_ in the presence of drug/normalized max F_340/380_ in the absence of drug) to Nitrendipine (1 μM; WT: *n* = 121; KO: *n* = 138), **(B)** ω-conotoxin G IVa (1 μM; WT: *n* = 222; KO: *n* = 219) and **(C)** ω-Agatoxin IVa (100 nM; WT: *n* = 213; KO: *n* = 249). The DR was compared in WT and *Fmr1*-KO with Mann-Whitney test: ****P < 0.0001; ns: *P* = 0.2969, not significant. These results are summarized in Table 2.

Highly specific pharmacological blockers have been identified for all these VGCC subfamilies (Zamponi et al., 2015). For instance, we used specific pharmacological blockers of VGCCs: dihydropyridines, such as nitrendipine, block L-type VGCCs (Peterson et al., 1996) by binding to transmembrane domains of the α1 subunit hence affecting the gating mechanism of the L-type VGCCs. ω-Conotoxin-GVIa (Conotoxin) blocks N-type VGCCs (Ichida et al., 2005) by interacting with the channel pore. ω-Agatoxin IVa (Agatoxin) inhibits P/Q-type VGCCs (Adams et al., 1993) by binding to two extracellular loops of the α1 subunit that are close to the sensor domain of the P/Q-channel. Thus, we used some of these blockers in order to further investigate the molecular determinants of such calcium homeostasis deregulations. Within each neuron expressing or not FMRP, we measured the DR as the ratio of the mean of the maximal depolarization-induced Ca^2+^ entry in the presence of a VGCC-specific antagonist on the mean calcium entry in the absence of a VGCC-specific antagonist. All the antagonists tested significantly reduced calcium ion entry upon KCl stimulation. Indeed, each antagonist treatment produced a DR that was statistically different from 1, the DR value expected for a drug having no effect (one sample *t*-test, *P* < 0.0001; Figures 2A–C). Nevertheless, Nitrendipine (1 μM) reduced KCl-triggered calcium ion entry similarly in WT and *Fmr1*-KO cells (Mann Whitney test, n.s.: *P* = 0.2968; Figure 2A). The ω-Conotoxin-GVIa (Conotoxin; 1 μM) was more efficient in *Fmr1*-KO cells (Mann Whitney test, *P* < 0.0001; Figure 2B). On the contrary, the ω-Agatoxin IVa (Agatoxin; 100 nM) had a fainter effect in *Fmr1*-KO than in WT cells (Mann Whitney test, *P* < 0.0001; Figure 2C). These findings strongly suggest that N- and P/Q-type channels are deregulated in *Fmr1*-KO neurons. These results are recapitulated in Table 2.

**Table 2:** Results summary.

	Mean ± SEM WT (*n*)	Mean ± SEM KO (*n)*	*P* value	*P* value significance
Figure 1D resting	−1.636 ± 0.016 (697)	−1.459 ± 0.0141 (744)	<0.0001	****
Figure 1E DHPG	0.3221 ± 0.0150 (222)	0.2989 ± 0.0105 (211)	0.9963	ns
Figure 1F KCl	1.297 ± 0.0142 (697)	1.154 ± 0.0147 (744)	<0.0001	****
Figure 1G After KCl	0.5509 ± 0.0054 (697)	0.5709 ± 0.0051 (744)	0.0038	**
Figure 2A Nitrendipine response	0.3497 ± 0.0215 (121)	0.3273 ± 0.0196 (138)	0.2968	ns
Figure 2B Conotoxin response	0.1088 ± 0.0087 (222)	0.1681 ± 0.0090 (219)	<0.0001	****
Figure 2C Agatoxin response	0.1478 ± 0.0085 (213)	0.0961 ± 0.0087 (249)	<0.0001	****

*Mann-Whitney test was used to assess statistical significance*.

### *Cacna1a* Expression Is Altered in *Fmr1*-KO Primary Neurons

**Figure 3:**
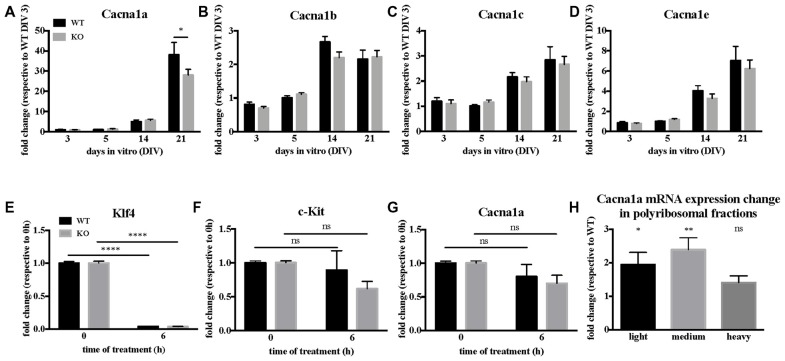
*Cacna1a* expression is deregulated in the absence of Fragile X Mental Retardation Protein (FMRP). **(A)** *In vitro* time-course of *Cacna1a*, **(B)** *Cacna1b*, **(C)** *Cacna1c* and **(D)** *Cacna1e* mRNA expression. Results are presented as the mean ± SEM, ANOVA two way Sidak’s multiple comparisons *post hoc* test: *P < 0.05. Quantification of *Klf4* **(E)**, *c-Kit* **(F)** and *Cacna1a* **(G)** mRNA levels upon actinomycin D treatment in Day-*In-Vitro* (DIV) 19–20 neuronal cultures. The mRNA levels of *c-Kit* as well as those of *Klf4* are used for comparison according to stability data from Sharova et al. (2009). Results are presented as the mean ± SEM, ANOVA two way with Sidak’s multiple comparisons test: ****P < 0.0001; ns, not significant (*c-Kit*: P_WT_ = 0.8386; P_KO_ = 0.0694. *Cacna1a*: P_WT_ = 0.4902; P_KO_ = 0.1071). **(H)** Quantification of *Cacna1a* mRNA relative expression levels (*Fmr1*-KO/WT) in light, medium and heavy polyribosomal fractions, respectively. Results are presented as the mean ± SEM, One-sample *t*-test: *P < 0.05; **P < 0.001; ns: *P* = 0.0717, not significant.

The pore forming unit of P/Q-type VGCC is encoded by the *Cacna1a* gene, whose mRNA is a target of FMRP (Darnell et al., 2011), in particular also during early brain development (at Post-Natal Day 13, PND 13; Maurin et al., 2018a). We therefore investigated how FMRP regulates *Cacna1a* expression in *Fmr1*-KO primary cultured neurons and in cortical extracts of *Fmr1*-KO mouse. We precisely characterized the time course of various α1 gene expression in WT and *Fmr1*-KO primary neurons by RT-qPCR. *Cacna1a* is the most upregulated α1 gene of the Ca_v_2 family between DIV 14 and 21, and its expression is reduced in *Fmr1*-KO neurons (Figures 3A–D) at DIV 21 compared to WT cells. We therefore investigated whether FMRP modulates *Cacna1a* mRNA half-life by measuring *Cacna1a* stability together with control RNAs in primary neurons treated with the polymerase II inhibitor Actinomycin D. We observed that, consistent with a previous report (Sharova et al., 2009), Actinomycin D treatment triggers a strong decrease in *Klf4* transcript expression (Figure 3E) which is not due to cell toxicity, as we could show that in the same conditions *c-Kit* expression is stable over time (Figure 3F). In these conditions, *Cacna1a* expression is affected to a similar extent in WT and *Fmr1*-KO neurons (Figure 3G), excluding a role of FMRP in regulating *Cacna1a* mRNA stability. We concluded that the decreased expression levels of *Cacna1a* mRNA in *Fmr1*-KO cells do not depend on the half-life of this mRNA in the absence of FMRP but it is likely due to a decreased transcription level. Thus, we analyzed *Cacna1a* translation in the cortex of WT and *Fmr1*-KO mice by quantifying *Cacna1a* mRNA levels in different fractions of polyribosome preparations obtained from WT and *Fmr1*-KO PND 13 mouse cortex. Our results show that *Cacna1a* mRNA polyribosome association is increased in the light and medium polyribosome fractions, which argues in favor of an increased translation of this mRNA in the absence of FMRP (Figure 3H).

**Figure 4:**
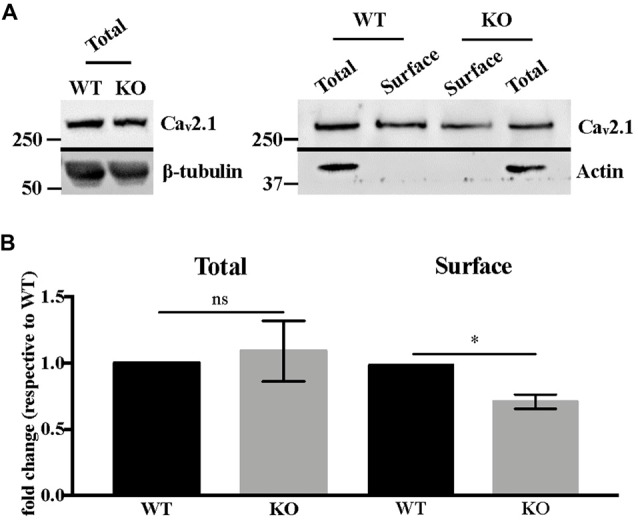
Ca_v_2.1 protein is mis-expressed at the plasma membrane of *Fmr1*-KO cortical neurons. **(A)** Western blot analysis of biotinylated Ca_v_2.1 in DIV 15–19 cortical neurons. β-tubulin is used as the loading control, whereas actin is used as the immunoprecipitation control. **(B)** Quantification of total and cell-surface Ca_v_2.1 protein levels. Results are presented as the mean ± SEM, Mann-Whitney test: *P < 0.05; ns: *P* = 0.7, not significant.

Western blot analysis of total Ca_v_2.1 protein levels in DIV 17–21 primary neurons showed no statistically significant difference between WT and *Fmr1*-KO cells (Mann-Whitney test, *P* = 0.7, not significant; Figures 4A,B). We also analyzed Ca_v_2.1 expression at the plasma membrane of *Fmr1*-KO and WT primary neurons by performing biotinylation assay. Our results show that Ca_v_2.1 protein is less expressed at the cell surface of *Fmr1*-KO neurons (Mann-Whitney test, *P* < 0.05; Figures 4A,B).

**Figure 5:**
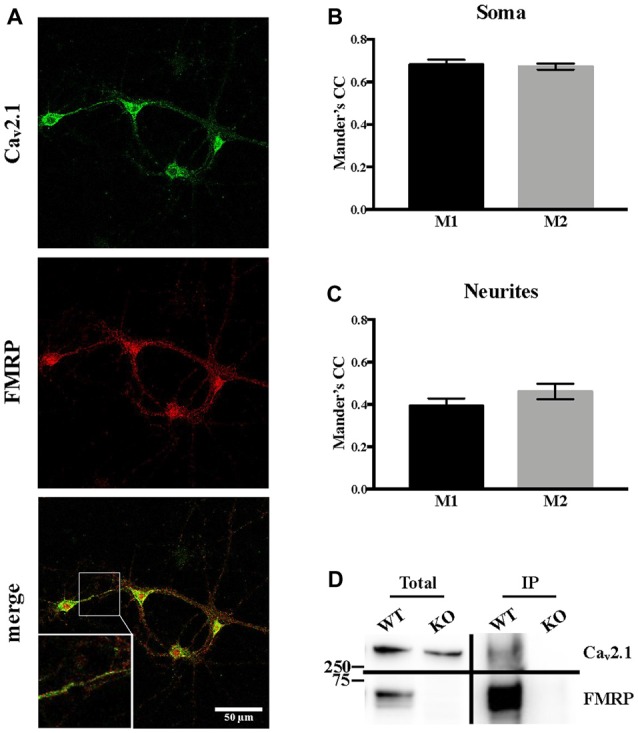
Endogenous Ca_v_2.1 interacts and partially co-localizes with FMRP. **(A)** Single plane confocal analysis of FMRP (revealed with the 1C3 antibody) and Ca_v_2.1 (revealed with the antibody anti-Ca_v_2.1) localization in DIV 13 primary neuronal cultures. The scale bar of each panel is 50 μm. **(B)** Quantification of the colocalization of FMRP and Ca_v_2.1 in the soma (*n* = 19) and **(C)** in neurites (*n* = 14) was performed with the JACoP plugin for ImageJ. CC, correlation coefficient; M1, fraction of FMRP overlapping with Ca_v_2.1; M2, fraction of Ca_v_2.1 overlapping with FMRP. **(D)** Endogeneous FMRP co-immunoprecipitation with Ca_v_2.1 in mouse cerebellar extracts. FMRP was revealed with the 1R antibody (Bonaccorso et al., 2015).

Since it was reported that, when overexpressed, FMRP directly interacts with both Ca_v_2.1 and Ca_v_2.2 (Ferron et al., 2014), we assessed whether Ca_v_2.1 and FMRP are colocalized in cortical neurons. Using double immunofluorescent staining and confocal microscopy, we observed and quantified their colocalization using Mander’s coefficients both in soma and in neurites (Figures 5A–C). These findings were also confirmed by biochemistry experiments performed on cerebellar extracts from PND 13 mice in which we showed that endogenous Ca_v_2.1 co-immunoprecipitates with FMRP (Figure 5D).

## Discussion

We and others have shown that among the FMRP mRNA targets many encode ion channels, sensors of intracellular ion concentration and other regulators of ion homeostasis (Brown et al., 2001; Darnell et al., 2011; Maurin et al., 2018a). Nonetheless, the direct interaction of FMRP with ion channels has been reported previously (Brown et al., 2010; Ferron et al., 2014; Myrick et al., 2015; Ferron, 2016). Also, it is not surprising that deregulations of expression levels as well as activities of ion channels have been shown in *Fmr1*-KO neurons (Chen et al., 2003; Meredith et al., 2007; Brown et al., 2010; Deng et al., 2013; Ferron et al., 2014; Zhang et al., 2014; Deng and Klyachko, 2016), some directly implicating VGCC deregulation in FXS (Chen et al., 2003; Meredith et al., 2007; Deng et al., 2013; Ferron et al., 2014; Zhang et al., 2014). Even if some of the conclusions of various studies were not completely convergent (Meredith et al., 2007; Ferron et al., 2014; Zhang et al., 2014), collectively these works suggest that the Ca^2+^ signaling-associated pathways may be involved in the physiopathology of FXS. For this reason, we decided to study calcium homeostasis in live, cultured neurons in the presence and in the absence of FMRP, using calcium imaging.

### FMRP Regulates VGCC Expression and Function

VGCCs play key roles in neurons, notably by regulating membrane excitability, neurotransmitter release and gene expression modulation (Simms and Zamponi, 2014). Alterations in the plasma membrane expression of these channels lead to pathological phenotypes, ranging from ataxia, ID, ASD and epilepsy (Yue et al., 1997; Damaj et al., 2015). Thus, to gain further insight in the Ca^2+^ pathway-associated molecular pathology in FXS, we carried out a pharmacological approach using VGCC-specific antagonists in our cellular model. We showed that both N- and P/Q-type VGCC inhibition differently affected KCl-mediated entry in WT and *Fmr1-*KO neurons. Indeed, blocking N-type VGCCs was more efficient in *Fmr1*-KO than in WT neurons and conversely, P/Q-type inhibition had less effect in *Fmr1*-KO neurons, suggesting that both Ca_v_2.2 and Ca_v_2.1 activities are deregulated in the absence of FMRP. Interestingly, *Cacna1a* mRNA is a target of FMRP in various brain regions (Maurin et al., 2018a) and here we show that:

1. The membrane levels of Ca_v_2.1 channels are reduced in *Fmr1*-KO neurons, consistent with the reduced sensitivity to P/Q-type VGCC inhibition with Agatoxin. Since the intracellular levels of Ca_v_2.1 do not appear to be altered (Figure 4), we conclude that Ca_v_2.1 direct interaction with FMRP could play a role in its function/localization in the absence of the partner. Also, the altered actin cytoskeleton organization described in different FXS cell lines (Castets et al., 2005; Nolze et al., 2013; Abekhoukh and Bardoni, 2014; Abekhoukh et al., 2017) may explain the reduced membrane expression of Ca_v_2.1, since cytoskeleton is the route for the correct subcellular localization of mRNAs (Bramham and Wells, 2007). It is worth reminding that altered sublocalization of membrane proteins (encoded by mRNA targets of FMRP) have been already described, such as diacylglycerol lipase-α (DGL-α; Jung et al., 2012), Homer 1 (Giuffrida et al., 2005; Aloisi et al., 2017) and Kv4.2, (Gross et al., 2011). Similarly, Ca_v_2.1 could be one of the deregulated elements. Interestingly, FMRP binds the mRNAs of other of its interacting proteins such as FMRP, CYFIP2, FXR1, Ca_v_2.2 (Darnell et al., 2011; Maurin et al., 2018a), suggesting a tight regulation of a FMRP-containing complex in a FMRP-dependent manner. Furthermore, the multiple mRNA targets of FMRP likely generate a network of interactions among FMRP-dependent pathways whose functional consequences are not easily predictable only considering the main role of FMRP as a translational repressor.
2. Even if the level of the mRNA encoding *Cacna1a* is slightly decreased in *Fmr1*-KO neurons at DIV21 (Figure 3A), the translational upregulation of this mRNA (as predicted by the increased polyribosome association of Ca_v_2.1 mRNA in *Fmr1*-KO brain compared with WT; Figure 3H) counterbalances the reduced mRNA level of *Cacna1a* in mature neurons. As in a yin-yang effect, this leads to unaltered total Ca_v_2.1 levels. We did not find any FMRP-dependent effect on RNA stability of *Cacna1a*, leading to the conclusion that the reduced level of *Cacna1a* mRNA in *Fmr1*-KO neurons is rather due to an indirect transcriptional deregulation.

### Pre-synaptic Calcium Channels in FXS and ASD

**Figure 6:**
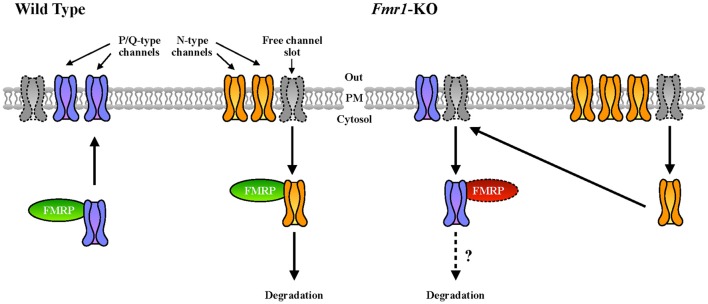
Our working model of FMRP-mediated regulation of VGCC developmental switch. In WT cells, N-type channels (in yellow), that are expressed first, are inserted in the plasma membrane and occupy most of the available N- and P/Q-preferring “channel slots” at the synapse (Cao et al., 2004; Cao and Tsien, 2010). We hypothesize that upon development and probably upon specific stimuli, FMRP could contribute to the replacement of N- by P/Q-type (in purple) VGCCs. In *Fmr1*-KO neurons, this replacement could be impaired resulting in an altered plasma membrane expression ratio between P/Q- and N-type channels.

Ca_v_2.2 was previously described to be more expressed and present at the plasma membrane of cells in the absence of FMRP (Ferron et al., 2014). This is consistent with the increased sensitivity to conotoxin that we observed in *Fmr1*-KO neurons compared to WT. At the molecular level, this abnormality was explained on the basis of the interaction (by overexpression) between FMRP and both Ca_v_2.2 and Ca_v_2.1 channels (Ferron et al., 2014). Interestingly, we confirmed here this latter finding by showing that the interaction between the endogenous proteins also occurs in brain (Figure 5D). Remarkably, we showed here that in *Fmr1*-KO cells Ca_v_2.1 expression deregulation is opposite to the one of Ca_v_2.2 (Ferron et al., 2014). As we already stated, FMRP also binds Ca_v_2.1 mRNA transcripts, indeed strongly suggesting a central role of FMRP in the regulation of P/Q- and N-type channels relative expression. Interestingly, it was shown that in cultured hippocampal synapses, P/Q- and N-type channels have preferred plasma membrane slots (Cao et al., 2004; Cao and Tsien, 2010) and according to this model, there are exclusive N-type channel slots and P/Q- preferring slots that can be used by N-type channels. For instance, in neurons expressing mutated P/Q-channels that lead to familial hemiplegic migraine type disease, N-type channel currents are increased, either by an increased release probability or rather by an increased N-type expression at the plasma membrane (Cao and Tsien, 2010). Collectively, these findings suggest that some P/Q-type channel slots can actually be occupied by N-type channels upon P/Q-type deficiency. Since FMRP has been shown previously to regulate N-type expression by targeting this channel to the proteasome (Ferron et al., 2014), it is tempting to speculate that FMRP is a molecular adaptor regulating the relative plasma membrane expression of N- and P/Q-type channels. In addition, by regulating the subcellular mRNA localization and/or translation of these channel types, it may also directly modulate their presence at the plasma membrane (Figure 6). Future studies will clarify the precise molecular mechanisms underpinning this deregulation in FXS, but it is interesting to underline here that an imbalance between the levels and the activities of N- and P/Q-type channels, could have some impacts on the physiopathology of FXS. Indeed, the differences in N- and P/Q-type inactivation kinetics, their various effects on short term plasticity (Inchauspe et al., 2004) and their different sensitivity to G-protein-coupled receptor-mediated inhibition of neurotransmitter release may have strong impacts on the functioning of synapses (Bourinet et al., 1996). Noteworthy, P/Q-type channel activity, but not N-type, mediates GABA release in fast spiking interneurons in rat pre-frontal cortex (Zaitsev et al., 2007). This suggests that abnormal GABA secretion at the temporoammonic branch of the perforant path in the *Fmr1*-KO mouse model (Wahlstrom-Helgren and Klyachko, 2015) could be related to Ca_v_2.1 expression defects. Furthermore, it was reported that the maximal inhibition by the GABAB receptor agonist baclofen was greater for EPSCs mediated by N-type channels than for those mediated by P/Q-type channels (Ishikawa et al., 2005). Consequently, in *Fmr1*-KO mice it is likely that the compensation of P/Q- by N-type channels have strong consequences on GABAB inhibition by weakening its effect on presynaptic release, likely leading to network hyper-excitability.

### Impairment of Calcium Homeostasis as a New Phenotype of *Fmr1*-KO Neurons. Is It a Novel Biomarker?

Implications of our findings are twofold, biological and clinical. Indeed, the FXS research field actively seeks new treatments and biomarkers to evaluate their efficiency (Castagnola et al., 2017; Maurin et al., 2018b) and, to date, the main cellular biomarker of cultured *Fmr1*-KO neurons is represented by their abnormal dendritic spine morphology, whose analysis requires exquisite expertise (Khayachi et al., 2018). Conversely, using spectroscopy, calcium concentration measurements can be routinely performed in most laboratory settings, making it an easy and robust marker to monitor drug efficacy. Here, we applied this technique to primary cultured neurons but it will also be possible to perform it in iPS-derived neurons thus obtaining, for the first time, a molecular marker that can be functionally quantified. This can be useful for diagnostic purposes and particularly as a follow-up for specific therapies. Indeed, the search for specific and easily measurable biomarkers for FXS as well as for ASD is urgent. For instance, since 2009 one of the conclusions of the Outcome Measures Working Groups for Fragile X was “…research on biomarkers for detecting treatment response in FXS was in its infancy, but this was an area of utmost importance” (Berry-Kravis et al., 2013). More recently, the accurate analysis of 22 double-blind controlled clinical trials in FXS finalized between 2008 and 2015 led to the conclusion that the readouts employed to evaluate the outcome of treatments were in general of moderate/poor quality (Budimirovic et al., 2017). Last but not least, this cellular biomarker could be used as the readout for screenings of small-molecule (singular) libraries (Bardoni et al., 2017) to define new treatments opportunities for FXS.

### Study Limitations

There are several limitations of this study that one may consider:

1. Our ImageJ macro analysis resulted in the identification of four types of cells, which is clearly underestimating the complexity of the cell population. We nevertheless trust that this approach will be useful to identify a cell type of interest in the future, associating morphological parameters with molecular/physiological determinants; interestingly, Ota et al. (2018) very recently published a study highlighting the benefits of identifying cells according to their shape;
2. In agreement with the expression levels of Ca_v_2.1, we focused our study on mature neuron cultures. This VGCC deregulation may not be observed in different culture settings;
3. The polyribosome fractionation experiments were performed on cortex extracts from PND 13 mice, preventing the identification of actively translating ribosomes through pharmacological inhibition. Therefore, we can only speculate that the increased presence of *Cacna1a* mRNA in light and medium fractions reflects an increased translation of this mRNA in *Fmr1*-KO mice;
4. The working model describing the putative role of FMRP in the regulation of N- and P/Q-type VGCCs at the plasma membrane (Figure 6) awaits a molecular mechanism and therefore is speculative. It nevertheless may be considered as a starting point for future analyses.

## Author Contributions

TM, FD and AF designed the experiments. SC, SD, AF, MB, MJ, MG and TM performed the experiments. FB designed and wrote the macro in ImageJ. AP performed the unsupervised multivariate analysis. TM, SC, AF, AP, MM, SM and BB analyzed the data. TM, SC and BB wrote the manuscript.

## Conflict of Interest Statement

The authors declare that the research was conducted in the absence of any commercial or financial relationships that could be construed as a potential conflict of interest.

## Supplementary Material

FIGURE S1 Cortical primary neuronal cultures show a negligible level of astrocytic growth. **(A)** Fluorescent analysis showing the level of GFAP (in green) in Day-In-Vitro (DIV) 12 WT primary neuronal cultures compared to the total number of cells (DAPI staining in blue for nuclei). **(B)** Percentage of GFAP-positive cells (20 imaged regions; *n* = 261 DAPI-positive cells; *n* = 23 GFAP-positive cells). **(C)** Quantification of *Gfap* and *Psd95* (*Dlg4* transcript) mRNA levels in DIV 20 cortical neurons (*n* = 3 independent cultures). Results are presented as the mean ± SEM, Mann-Whitney test: ns, not significant (*Gfap*: *P* = 0.3701; *Psd95*: *P* = 0.6200).

FIGURE S2 Unsupervised analysis of the shape and calcium homeostasis parameters of primary neuron cultures leads to the identification of four different groups of Regions-of-Interest (ROIs). Shape and calcium homeostasis parameters were first visualized in 2-dimension space using t-Distributed Stochastic Neighbor Embedding (t-SNE), then K-means clustering was performed on the 2-dimension t-SNE projection, and the optimal number of clusters, was determined using the Gap statistic. **(A)** t-SNE representation of the data, with cells colored by genotype. The distribution of WT (black dots) and *Fmr1*-KO (red dots) is homogeneous and vastly overlapping in all clusters. **(B)** t-SNE representation of the data, with cells colored by cluster. The distribution of the parameters of interest by clusters are presented using boxplots. The boxplots are defined as 25th percentile–75th percentile, the horizontal line corresponds to the median value, and whiskers extend to the min-max values. **(C)** Area covered by the cells, **(D)** cell circularity, **(E)** cell solidity, **(F)** cell roundness, **(G)** cell resting intracellular calcium concentration and **(H)** maximal KCl-triggered intracellular calcium concentration. **(I–L)** Number of cells from each genotype in the various experiments: ω-agatoxin-IVa (Aga), ω-conotoxin GVIa (Cono), Nitrendipine (Nitren) or in the absence of VGCC antagonist (NoDrug) show the homogeneous WT and *Fmr1*-KO cell distribution in all identified clusters.

FIGURE S3 Representative images of ROIs identified using the ImageJ macro. Left panels are pseudo-colored images of stabilized unstimulated cells **(a, d, g, j)**. Middle panels represent the same cells during KCl stimulation **(b, e, h, k)**. Right panels show the macro output result **(c, f, i, l)**. The ROIs are encircled by a yellow line and the numbers indicate to which cluster ROI were attributed.

FIGURE S4 The percentage of DHPG-responding cells is similar in WT and *Fmr1*-KO neurons. Cells in which the pharmacological stimulation elicited at least a 1.1 fold change in the F_340/380_ ratio compared to baseline F_340/380_ were considered DHPG-responsive and were counted in each cell cluster.
