# Supplementary figures and images for: New Insights Into the Role of Cav2 Protein Family in Calcium Flux Deregulation in Fmr1-KO Neurons
Source: Front Mol Neurosci. 2018 Sep 27;11:342. doi: 10.3389/fnmol.2018.00342 (PMC6170614; doi:10.3389/fnmol.2018.00342)

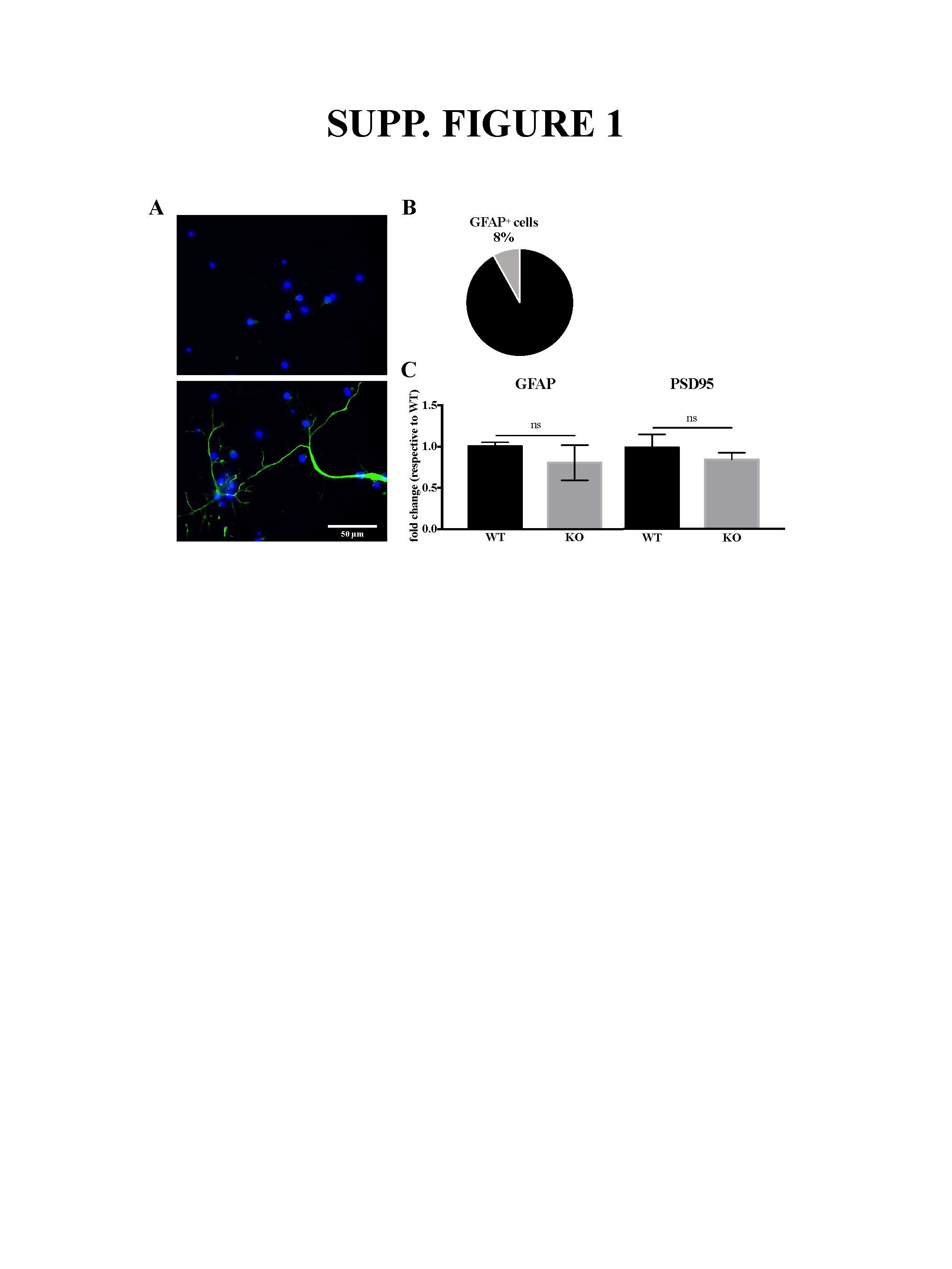

Supplement: FIGURE S1 — Cortical primary neuronal cultures show a negligible level of astrocytic growth. (A) Fluorescent analysis showing the level of GFAP (in green) in Day-In-Vitro (DIV) 12 WT primary neuronal cultures compared to the total number of cells (DAPI staining in blue for nuclei). (B) Percentage of GFAP-positive cells (20 imaged regions; n = 261 DAPI-positive cells; n = 23 GFAP-positive cells). (C) Quantification of Gfap and Psd95 (Dlg4 transcript) mRNA levels in DIV 20 cortical neurons (n = 3 independent cultures). Results are presented as the mean ± SEM, Mann-Whitney test: ns, not significant (Gfap: P = 0.3701; Psd95: P = 0.6200). [file Image_1.TIFF]

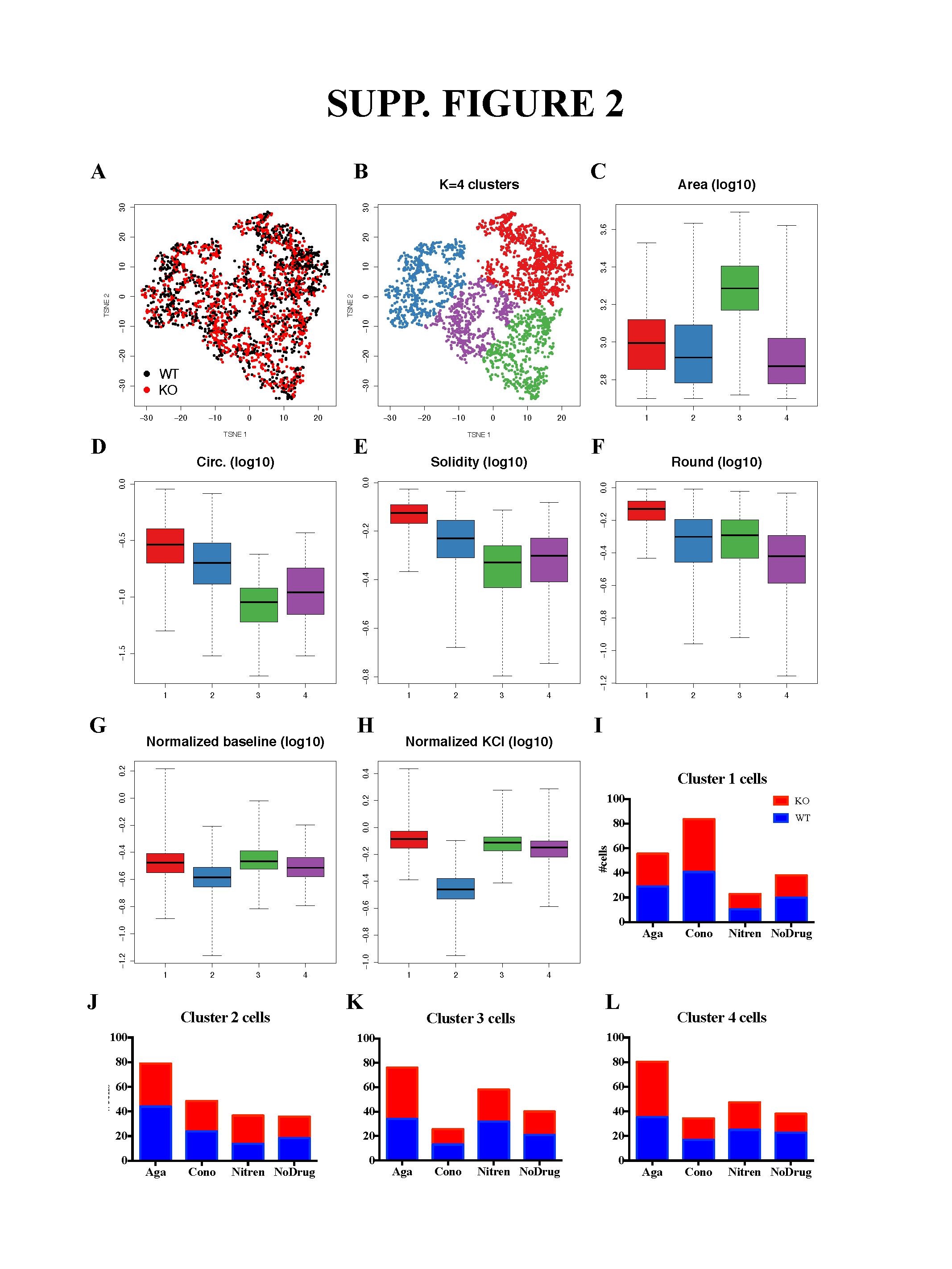

Supplement: FIGURE S2 — Unsupervised analysis of the shape and calcium homeostasis parameters of primary neuron cultures leads to the identification of four different groups of Regions-of-Interest (ROIs). Shape and calcium homeostasis parameters were first visualized in 2-dimension space using t-Distributed Stochastic Neighbor Embedding (t-SNE), then K-means clustering was performed on the 2-dimension t-SNE projection, and the optimal number of clusters, was determined using the Gap statistic. (A) t-SNE representation of the data, with cells colored by genotype. The distribution of WT (black dots) and Fmr1-KO (red dots) is homogeneous and vastly overlapping in all clusters. (B) t-SNE representation of the data, with cells colored by cluster. The distribution of the parameters of interest by clusters are presented using boxplots. The boxplots are defined as 25th percentile–75th percentile, the horizontal line corresponds to the median value, and whiskers extend to the min-max values. (C) Area covered by the cells, (D) cell circularity, (E) cell solidity, (F) cell roundness, (G) cell resting intracellular calcium concentration and (H) maximal KCl-triggered intracellular calcium concentration. (I–L) Number of cells from each genotype in the various experiments: ω-agatoxin-IVa (Aga), ω-conotoxin GVIa (Cono), Nitrendipine (Nitren) or in the absence of VGCC antagonist (NoDrug) show the homogeneous WT and Fmr1-KO cell distribution in all identified clusters. [file Image_2.TIFF]

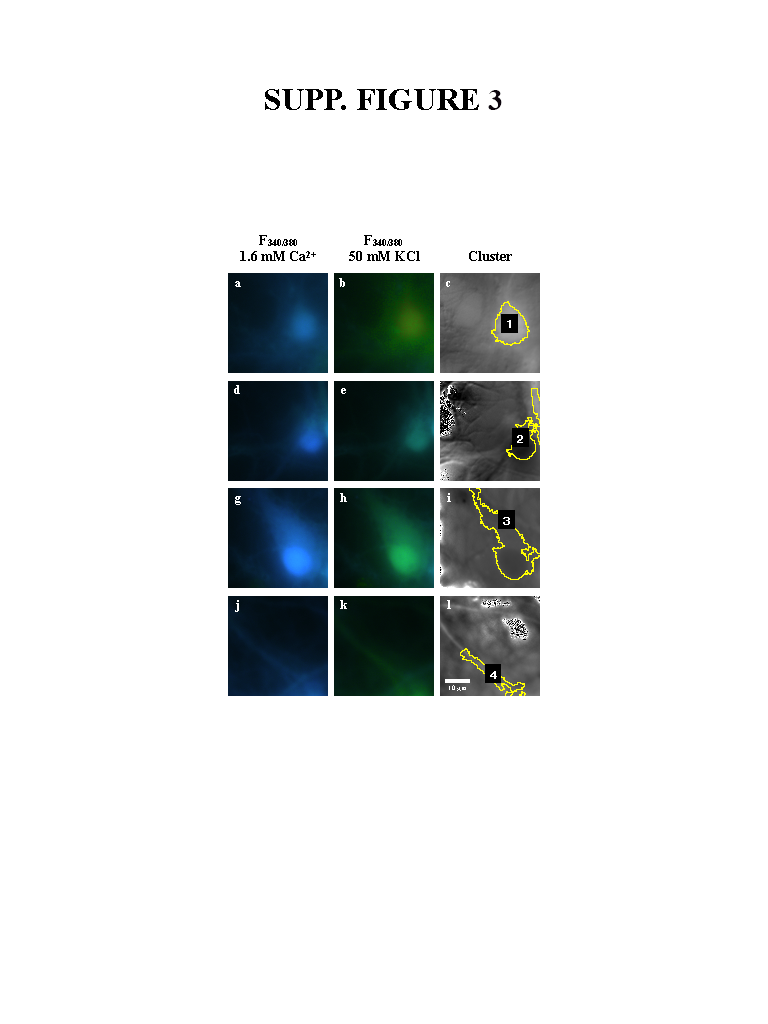

Supplement: FIGURE S3 — Representative images of ROIs identified using the ImageJ macro. Left panels are pseudo-colored images of stabilized unstimulated cells (a, d, g, j). Middle panels represent the same cells during KCl stimulation (b, e, h, k). Right panels show the macro output result (c, f, i, l). The ROIs are encircled by a yellow line and the numbers indicate to which cluster ROI were attributed. [file Image_3.tif]

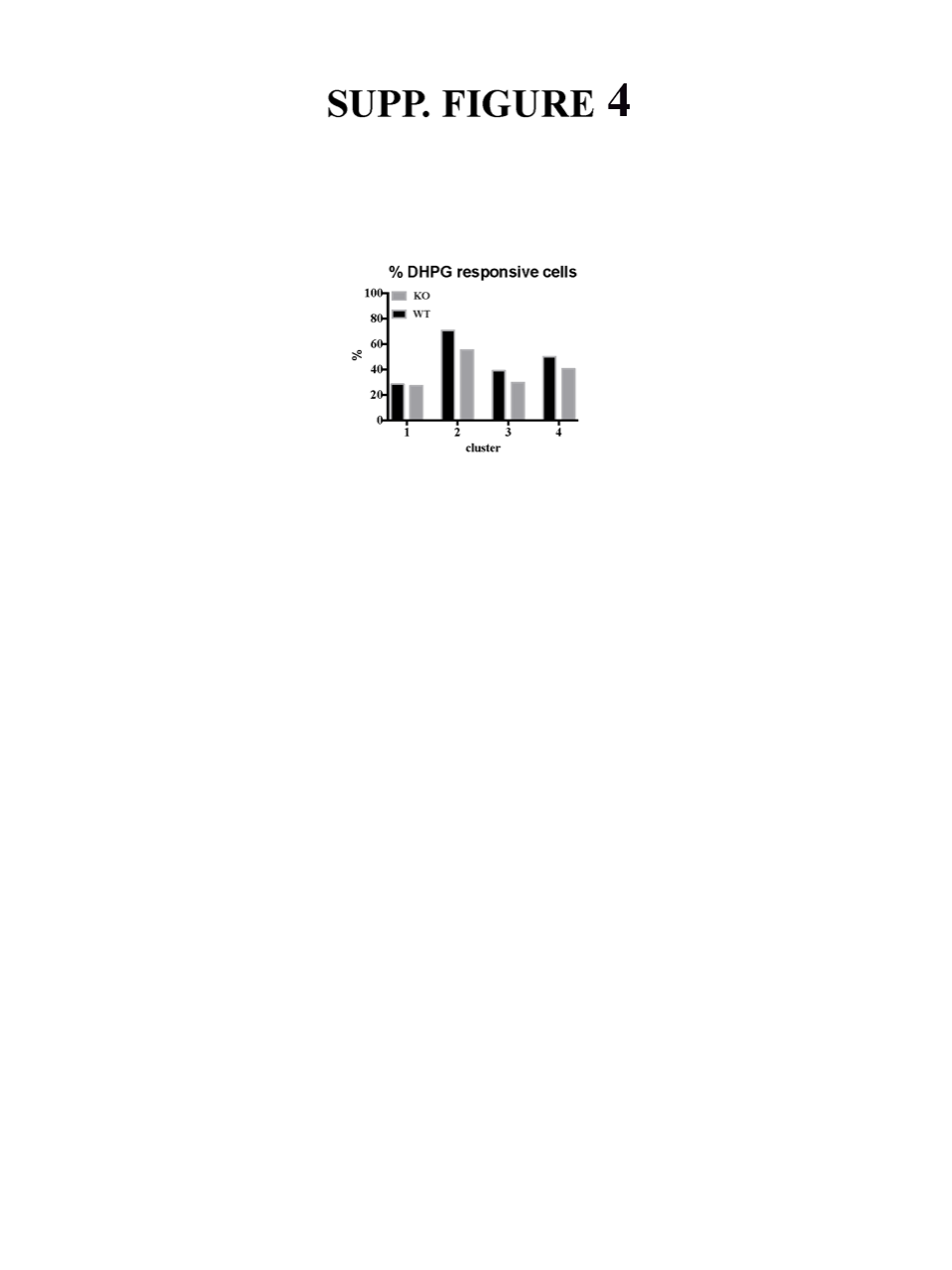

Supplement: FIGURE S4 — The percentage of DHPG-responding cells is similar in WT and Fmr1-KO neurons. Cells in which the pharmacological stimulation elicited at least a 1.1 fold change in the F340/380 ratio compared to baseline F340/380 were considered DHPG-responsive and were counted in each cell cluster. [file Image_4.tif]
